# Safety, Efficacy and Pharmacokinetics of a New 10% Liquid Intravenous Immunoglobulin (IVIG) in Patients with Primary Immunodeficiency

**DOI:** 10.1007/s10875-012-9656-5

**Published:** 2012-03-06

**Authors:** Richard L. Wasserman, Joseph A. Church, Mark Stein, James Moy, Martha White, Steven Strausbaugh, Harry Schroeder, Mark Ballow, James Harris, Isaac Melamed, David Elkayam, William Lumry, Daniel Suez, Syed M. Rehman

**Affiliations:** 1 Pediatric Allergy/Immunology Associates, 777 Forest Lane, Suite B-332, Dallas, TX 75230 USA; 2 Children’s Hospital Los Angeles, Los Angeles, CA USA; 3 Allergy Associates of Palm Beaches, North Palm Beach, FL USA; 4 Rush Universty Medical Center, Chicago, IL USA; 5 Institute for Allergy and Asthma, Wheaton, MD USA; 6 University Hospital Case Medical Center, Cleveland, OH USA; 7 University of Alabama at Birmingham, Birmingham, AL USA; 8 Women & Children’s Hospital of Buffalo, Buffalo, NY USA; 9 South Bend Clinic LLP, South Bend, IN USA; 10 1st Allergy & Clinical Research Center, Centennial, CO USA; 11 Bellingham Asthma, Allergy & Immunology Clinic, Bellingham, WA USA; 12 Allergy and Asthma Specialists, Dallas, TX USA; 13 Allergy, Asthma & Immunology Clinic, PA, Dallas, TX USA; 14 Allergy & Asthma Center, Toledo, OH USA

**Keywords:** Intravenous immunoglobulin (IVIG), primary immunodeficiency (PID), clinical trial, safety, efficacy, pharmacokinetics

## Abstract

**Introduction:**

An investigational 10% liquid intravenous immunoglobulin (IVIG) was studied in 63 patients with primary immunodeficiency (PID) at 15 study sites.

**Methods:**

Patients were treated every 3 or 4 weeks with 254–1029 mg/kg/infusion of IVIG.

**Results:**

Overall, Biotest-IVIG infusions were well tolerated. The proportion of infusions that were associated with adverse events during infusion, and up to 72 h after infusion, including those unrelated to study product, was 27.7% with an upper 95% confidence limit ≤30.6%. Two serious bacterial infections (SBIs) were observed resulting in a serious bacterial infection rate of 0.035 per person per year and an upper one-sided 99% confidence limit of ≤0.136 SBI/patient/year. The number of days of work or school missed due to infection were relatively low at 2.28 days/patient/year. Two patients were hospitalized for infection producing a rate of 0.21 hospitalization days/patient/year. The IgG half-life was approximately 30 days with variation among individuals.

**Conclusions:**

Pharmacokinetic parameters of specific antibody activities were essentially the same as those of total IgG. Biotest-IVIG is safe and effective in the treatment of PID.

## Introduction

Human immunoglobulin G (IgG) has been used to treat people with inherited antibody deficiencies since 1952 when Bruton demonstrated that monthly subcutaneous injections of IgG successfully prevented recurrent pneumococcal infections in a child with agammaglobulinemia [1]. The first commercial human IgG preparations were restricted to intramuscular injection at relatively low doses, usually 25 mg/kg/week. In order to avoid the pain of intramuscular IgG injections and to administer larger doses, intravenous immunoglobulin products (IVIG) were developed. Periodic reports of hepatitis transmission and recognition that primary immunodeficient (PID) patients must be treated for a lifetime resulted in development of IVIG manufacturing procedures that inactivate or remove blood-borne pathogens. Manufacturing steps that enhance purity, minimize damage to IgG molecules, decrease the frequency of adverse reactions, and result in higher concentration liquid IVIG have also been introduced. We report here the results of a clinical trial of a new liquid 10% IVIG (Biotest-IVIG) in patients with primary immunodeficiency.

## Methods

### Study Product

The investigational 10% liquid IVIG used in this study (Biotest-IVIG) was manufactured by Nabi Biopharmaceuticals, subsequently acquired by Biotest Pharmaceuticals Corporation, in Boca Raton, Florida. Biotest-IVIG was manufactured from source plasma, fully screened for blood borne pathogens, by Cohn-Oncley fractionation followed by ion exchange chromatography [2, 3]. Three virus removal/inactivation steps (cold ethanol fractionation, solvent/detergent inactivation and nanofiltration) are incorporated into the manufacturing process [4–8]. The final product contains 100 ± 10 mg/mL protein, sodium chloride, glycine and polysorbate 80 at low pH. The IgG monomer plus dimer content is >95%.

### Study Patients

Sixty-three patients with primary immunodeficiency were enrolled and treated at 15 study sites. Enrollees were male or female ages ≥ 6 and ≤ 75 years with a confirmed diagnosis of primary immunodeficiency, a history of hypogammaglobulinemia (i.e., IgG < 500 mg/dL) or deficient antibody production prior to IgG replacement therapy. Prior to enrollment, patients were required to have received IVIG infusions every 3 or every 4 weeks for ≥3 months at a dose aimed to be between 300 and 800 mg/kg. Patients were excluded if they were positive at screening for any markers of infectious blood-borne viruses, had a history of adverse reactions to other IgG or blood products, had selective IgA deficiency or antibodies to IgA, had a history of acute renal failure/or severe renal impairment, had a history of deep venous thrombosis or were pregnant or lactating.

### Study Design

This was an open-label, phase III safety, efficacy and pharmacokinetic study of patients with documented primary immunodeficiency. Each patient was scheduled to receive a Biotest IVIG infusion of 300 to 800 mg/kg every 3 or 4 weeks for approximately 1 year. Dosing intervals were determined by the patient’s pre-study dosing schedule. All patients had safety monitoring studies performed prior to each infusion, at the final clinical visit and 3 months after the last infusion. Blood samples were collected before each infusion for determination of trough IgG levels. Patients also had blood samples taken prior to several infusions to test for anti-*Streptococcus pneumoniae*, anti-*Haemophilus influenzae* type b and anti-tetanus toxoid antibody concentrations.

Twenty patients (5 on the 3 week and 15 on the 4 week dosing schedule) were enrolled in the pharmacokinetic (PK) portion of the study. Tests for total IgG, IgG subclasses, *Streptococcus pneumoniae* antibodies, *Haemophilus influenzae* type b antibody and tetanus toxoid antibody were performed for PK analysis.

When necessary, doses of Biotest IVIG were adjusted during the study to maintain minimum trough IgG concentrations >500 mg/dL.

### Evaluation of Safety

Patients were monitored for adverse events (AEs) and serious adverse events (SAEs) during infusion and between infusions using home diaries. AEs were defined as a treatment-emergent adverse events associated with the use of IVIG, whether or not the AE was determined to be product related. The patients recorded all AEs, complaints or problems including start and stop dates, start and stop times and severity in their diaries.

AEs occurring during and within 72 h of infusion regardless of causality were considered temporally associated adverse events (TAAE). The primary safety endpoint was defined as the proportion of infusions with ≥1 temporally associated AEs including those that were determined to be unrelated to the investigational product. The target for this endpoint was an upper one-sided 95% confidence limit of less than 0.40 [9].

### Evaluation of Efficacy

The primary efficacy endpoint was demonstration that the rate of acute serious bacterial infections (SBIs) was less than 1.0 per person-year during regular administration of investigational IVIG for 12 months. Diagnostic criteria for SBIs were defined prospectively [9].

Secondary efficacy parameters included all infections of any kind or severity, time to the first infection of any kind, time to first SBI, days missed from school or work due to infection, days on antibiotics, hospitalizations and days of hospitalization due to infection.

### Evaluation of Pharmacokinetics (PK)

PK assessments were performed at the 4^th^ or 5^th^ infusion of study IVIG in order to wash out previous IVIG products. At certain study sites, PK parameters were assessed at infusion 13 or infusion 17 for patients who did not participate in the earlier PK study. Blood samples for PK assessment were taken before the infusion and at the following times after the infusion: 15 min, 1 h, 24 h, 3 days, 7 days, 14 days, 21 days and 28 days (if applicable.) The samples were tested for concentrations of total IgG, IgG subclasses and specific antibodies against several *S. pneumoniae* capsular polysaccharide serotypes, *H. influenzae* type b and tetanus toxoid. The calculated pharmacokinetic parameters were Cmax, the maximum serum concentration, Tmax, the time to reach the maximum serum concentration, AUC 0-t, the area under the concentration-time curve over 1 dosing interval, t_1/2_ or the elimination half-life, CL the total body clearance and Vz, the volume of distribution.

### Statistical Analysis

Descriptive summaries are provided where appropriate for each of the primary and secondary endpoints. In general, summaries are provided for the entire Safety, Intent to Treat (ITT), Per Protocol (PP) or PK populations and by IVIG infusion schedule (i.e., 3-week or 4-week dosing schedule). Continuous, quantitative, variable summaries include the number of patients (N), mean, standard deviation (SD), median, and range (minimum and maximum). Categorical, qualitative, variable summaries include the frequency and percentage of patients who are in the particular category. In general the denominator for percentage calculations was based upon the number of patients by infusion schedule (i.e., 3-week or 4-week-cycle) or overall, unless otherwise specified.

All efficacy and safety analyses were performed using SAS® Software version 8.2 or later. All pharmacokinetic parameter calculations were performed using WinNonlin Professional® version 5.2 or later but summary statistics were supported by SAS® Software version 8.2 or later.

## Results

Sixty-three patients were enrolled in this study, were treated with Biotest-IVIG and were included in the population evaluated for safety. Seventeen patients were infused with investigational product every 3 weeks and 46 were infused every 4 weeks. Of the 63 enrollees, 5 lost to follow-up when a study site was discontinued. This resulted in 58 patients in the ITT population. Seven patients with major protocol violations were excluded from the ITT population resulting in a per-protocol (PP) population of 51 patients. The ITT and PP populations were analyzed for efficacy.

As shown in Table I, 98% of patients were Caucasian and the most common immunodeficiency diagnosis was common variable immunodeficiency (CVID) (81% of patients). The average age was 41.2 years (range 6–75). Eight patients had a history of acute serious bacterial infection (SBI) prior to entry into this study. The most common was bacterial pneumonia in 7 patients.

**Table I:** Demographics and baseline characteristics of the safety population

Parameter	Total (*N* = 63)
Gender	
Female	32 (50.8%)
Male	31 (49.2%)
Age (yr)	
Mean (SD)	41.2 (19.68)
Median	44.0
Minimum, Maximum	6, 75
Age group	
6–11 Years	4 (6.3%)
12–17 Years	6 (9.5%)
18–64 Years	44 (69.8%)
65 Years and Older	9 (14.3%)
Race	
Caucasian	62 (98.4%)
Asian	1 (1.6%)
Primary diagnosis	
X-linked agammaglobulinemia	6 (9.5%)
Common variable immunodeficiency	51 (81.0%)
Other hypogammaglobulinemia	6 (9.5%)
SBI history	8 (12.7%)
Bacterial pneumonia	7 (11.1%)
Other	1 (1.6%)

### Safety

During the study a total of 746 infusions of Biotest-IVIG were administered to the 63 patients in the safety population with a mean of 13 infusions per patient and a range of 1 to 17 infusions. The mean dose per patient per infusion was 500 mg/kg with a range of 254 to 1029 mg/kg. Fifty-two patients were treated with study product for 12 months. More than 80% of patients were infused at a rate ≥ 3.0 mL/kg/h (300 mg/kg/h) and 49.6% were infused at a rate ≥ 3.5 mL/kg/h (350 mg/kg/h).

A total of 937 AEs were recorded in the 63 patients in the safety population (Table II). Of the 937 AEs, the most frequent (>20%) were headache (50.8%), sinusitis (38.1%) fatigue (28.6%), upper respiratory tract infection (25.4%), cough (22.2%), pharyngolaryngeal pain and diarrhea (each 20.6%), Six patients (9.5%) reported 339 (36.2%) of the 937 AEs.

**Table II:** Summary of adverse events in the safety population (*N* = 63)

Metric	Total (%)
Adverse events	937
Total number of infusions	746
Patients with ≥ 1AE	59 (93.7%)
Drug-related AEs	300 (32.0%)
Patients with ≥1 drug-related AE	40 (63.5%)
Infusions with ≥1 TAAE	209 (28.0%)
Infusions with ≥1 drug-related TAAE	152 (20.4%)
Patients with ≥1 SAE	7 (11.1%)
Number of SAEs	11
Patients with ≥1 drug-related SAE	1 (1.6%)
Patients with ≥1 AE leading to withdrawal	2 (3.2%)
Severity of drug-related AEs	
Mild (No. of patients)	11 (17.5%)
Moderate (No. of patients)	21 (33.3%)
Severe (No. of patients)	8 (12.7%)

*TAAE* temporally associated adverse event, i.e. occurs within 72 h of an infusion

*SAE* serious adverse event

Fifty-nine patients (93.7%) reported at least one adverse event. Of these, 40 patients (63.5%) were judged to have experienced an AE that was related to study product. Mild AEs occurred in 11 (17.5%) patients, moderate AEs occurred in 21 (33.3%) patients and severe AEs were reported in 8 (12.7%) patients. The most frequent severe, drug-related AEs were headache (3 patients, 4.8%), migraine and fatigue (2 patients, 3.2% each).

Eleven serious adverse events (SAEs) were reported in 7 (11.1%) patients. Two of the SAEs (vomiting, mild in severity and dehydration, moderate in severity) leading to hospitalization in 1 patient were considered as related to study product. None of the SAEs resulted in a dose change, dose interruption or discontinuation from the study and all the SAEs resolved. There were no deaths.

There were 431 temporally associated AEs (TAAEs) in 47 (74.6%) patients. Most TAAEs (335, 78%) occurred during and within the first 24 h after infusion in 36 (57.1%) patients. The proportion of infusions with ≥1 temporally associated AEs, regardless of relationship to study product, was 27.7% with an upper one-sided 95% confidence limit of ≤30.6%. This is significantly below the upper one-sided 95% confidence limit of 40% for TAAEs recommended by FDA [9]. The most frequent TAAE reported was headache (occurred with 115 infusions in 27 patients) followed by fatigue (59 infusions in 15 patients) (Table III).

**Table III:** Temporally associated adverse events (TAAEs) in more than 5% patients in the safety population (*N* = 63)

AE	By Patient (n,%) (47 patients)	By Infusion (n,%) (431 infusions)
Headache	27 (42.9%)	115 (15.4%)
Fatigue	15 (23.8%)	59 (7.9%)
Infusion site reaction	5 (7.9%)	5 (0.7%)
Nausea	5 (7.9%)	8 (1.1%)
Blood Pressure increased	4 (6.3%)	5 (0.7%)
Diarrhea	4 (6.3%)	4 (0.5%)
Dizziness	4 (6.3%)	4 (0.5%)
Lethargy	4 (6.3%)	4 (0.5%)

There was no clinically relevant effect of study treatment on systolic blood pressure or diastolic blood pressure measured before or after study infusions. Although several patients had AEs related to vital signs, no safety concerns were identified by the investigators.

There were no hematology or clinical chemistry abnormalities determined by an investigator to be clinically relevant. None of the patients exhibited signs of hemolysis at any time during the study as demonstrated by stable values of hematocrit, hemoglobin and LDH.

Tests for parvovirus B19, HIV, HCV and HBV were performed at screening, prior to infusions 8 and 12 and at the final safety follow-up visit. There was no evidence of viral transmission by study product. There was a single positive finding for parvovirus B19 during the study that was attributed to exposure to a child with symptomatic Fifth’s disease.

### Efficacy

Two serious bacterial infections occurred during the study. A 20 year old man treated every 4 weeks with study IVIG developed pneumonia 16 days after his last study infusion. He was hospitalized and the diagnosis of pneumonia was confirmed by computed axial tomography. The pneumonia resolved 5 days after diagnosis. A 48 year old woman treated every 4 weeks with study IVIG was hospitalized for apparent acute exacerbation of chronic obstructive bronchitis that was later classified by the FDA as bacterial pneumonia. These two episodes resulted in a serious bacterial infection rate of 0.035 SBI/patient/year with an upper 99% confidence limit of ≤0.136 SBI/patient/year. This result is substantially below the target SBI rate of ≤1.0 SBI/patient/year defined by the FDA as an appropriate endpoint for PID patients receiving regular doses of IVIG [9].

Infections of any kind or seriousness are listed in Table IV. Since the incidence of infections was essentially the same in the PP population as in the ITT population, the most frequent infections (other than SBI) were reported for the ITT population (Table V) and included acute sinusitis, other respiratory tract infections, unclassified infections designated as “other,” otitis media, and bronchitis.

**Table IV:** Infections of any kind or seriousness in the ITT and PP populations

Parameter	ITT (*N* = 58)	PP (*N* = 51)
Total Infections	139	132
Mean ± SD per subject	2.4 ± 2.7	2.6 ± 2.7
Infections per subject per year (90% CI)	2.6 [2.3–2.7]	2.6 [2.2–3.0]

**Table V:** Infections observed in the ITT population (*N* = 58)

Infections	No. of Patients (% of infections)
Acute sinusitis	36 (25.9%)
Other respiratory tract infection	30 (21.6%)
Other	23 (16.5%)
Otitis Media/ear infection	15 (10.8%)
Bronchitis	14 (10.1%)
Acute exacerbation of chronic sinusitis	7 (5.0%)
GI Infection	7 (5.0%)
Urinary tract infection	4 (2.9%)
Pneumonia	2 (1.4%)
Conjunctivitis	1 (0.7%)

Table VI lists the secondary efficacy endpoints observed in the ITT population. Means, standard deviations and rates are summarized. Twenty-one patients (33.3%) missed work or school because of infection at a rate of 2.28 days/patient/year (Table VI). Thirty-eight patients (60.3%) were treated with therapeutic antibiotics at a rate of 39.1 days of therapeutic antibiotics/patient/year. The rate of hospitalizations was 0.21 days per patient per year.

**Table VI:** Secondary efficacy endpoints in the ITT population

Parameter	Total (*N* = 58)
Infections of any kind (total)	139
Mean ± SD	2.4 ± 2.7
Min–Max	0–14
Patients with any infection (n)	40
Infection rate/patient/year	2.6
Days off work/school due to infection (n)	
Mean ± SD	2.1 ± 4.84
Min - Max	0–24
Patients with any days off work or school (n)	21
Days off work or school/patient/year	2.28
Days on antibiotics	
Mean ± SD	76.4 ± 118.3
Min - Max	0–372
Patients with any days on antibiotics (n)	46
Days of antibiotics/patient/year	82
Days on therapeutic antibiotics	
Mean ± SD	36.2 ± 52.7
Min - Max	0–306
Patients on therapeutic antibiotics (n)	38
Days of therapeutic antibiotics/patient/year	39.1
Days of hospitalization due to infection (n)	11
Patients hospitalized (n)	2
Hospitalization days/patient/year	0.21

*SD* standard deviation

### Pharmacokinetics

The PK population was composed of 5 patients in the 3-week infusion group and 16 in the 4-week group. PK parameters for total IgG are shown in Table VII. Cmax and Tmax were similar for the 2 treatment groups. The area under the concentration-time curve AUC _0-t_ was higher in 4-week patients than 3-week patients because of the longer time interval between infusions. The IgG half-life in 3-week subjects was 19.6 days compared to 33.5 days in 4-week subjects; however these differences were not significant.

**Table VII:** IgG pharmacokinetic parameters

Parameter	3-week cycle (*N* = 5) Mean ± SD)	4-week cycle (*N* = 16) Mean ± SD)
C_max_ (mg/dL)	2184 ± 293	2122 ± 425
T_max_ median (range) (h)	4.05 (2.67–26.1)	3.48 (2.6–78.6)
AUC 0-t (h*mg/dL)	668,173 ± 118,198	852,213 ± 155,334
T_1/2_ (days) (min-max)	19.6 (4.1)	33.5 (10.7)
	(16.2–26.7)	(18.3–51.6)
CL (mL/kg/day)	1.97 ± 0.22	1.41 ± 0.46
Vz (L/kg)	0.056 ± 0.014	0.064 ± 0.015

Pharmacokinetic parameters of specific antibodies were comparable to values for total IgG. Data from 28-day patients are shown in Table VIII. Similar results were observed in the five 21 day patients (data not shown). The antibody half-lives are the same order of magnitude as total IgG with values ranging from 25 to 84 days.

**Table VIII:** Pharmacokinetic parameters of specific antibodies in patients infused every 4 weeks

	Tetanus (*N* = 16) (IU/mL)	*H. influenzae* b (*N* = 15) (mg/L)	*S. pneumoniae* serotypes (μg/mL) (*N* = 16)		
			14	19A	23F
Elimination half-life (days)	83.76 (*n* = 14)	25.16^a^ (*n* = 14)	40.77 (*n* = 15)	60.04 (*n* = 15)	29.98 (*n* = 14)
Cmax (units)	6.96	6.62	14.62	46.14	36.38
Tmax (days)	0.131	0.644	1.821	1.861	1.682
AUC _0-t_ (units*days)	142.48 (*n* = 14)	96.09 (*n* = 14)	253.99 (*n* = 15)	1026.63 (*n* = 15)	563.89 (*n* = 15)

^a^Outlying value of patient 6003 was excluded from calculation.

The doses administered in this study produced mean trough IgG levels of 1076 ± 254 mg/dL (range 606 to 1780 mg/dL) in 21-day patients and 943 ± 215 (range 487 to 2250 mg/dL) in 28-day patients. The 487 mg/dL value was a single test result that was reported at the 6^th^ infusion in one patient; however, trough IgG levels above 500 mg/dL were observed at all other infusions in this patient.

## Discussion

Biotest-IVIG incorporates the best practices in IVIG manufacturing by combining Cohn-Oncley cold ethanol fractionation, purification by ion-exchange chromatography, solvent-detergent virus inactivation, virus removal by nanofiltration and formulation at 10% protein [2–8].

The purpose of this clinical trial was to evaluate the safety, efficacy, tolerability and pharmacokinetics of Biotest-IVIG in patients with primary immunodeficiency. The study trial followed the FDA guidelines published in 2008 that has lead to the standardization of many safety, efficacy and pharmacokinetic endpoints [9].

The observed types, severity and frequency of AEs assessed as medically related to Biotest-IVIG are commonly reported as associated with infusions of any IVIG. Eighty percent of the adverse events related to study IVIG were judged to be mild or moderate. As reported in other IVIG clinical trials [10–15], the most common adverse event was headache. A large proportion of adverse events (339, 36.2%) occurred in a small number of patients (6 patients, 9.5%). Although sporadic fluctuations in blood pressure and pulse were noted throughout the study, none were considered to be clinically significant. Analyses of serum chemistries, urinalysis, and viral safety tests showed no clinically significant or unexpected results for a population of PID patients receiving IVIG.

The primary efficacy endpoint, 0.035 SBI/person/year, compares favorably to SBI rates observed in studies of other IVIGs in patients with primary immunodeficiency who received IVIG doses that were similar to those administered in this study [10–15].

Infections other than serious infections occurred at a rate of 2.6 infections/patient/year. The most frequent infections were acute sinusitis and other respiratory tract infections.

Among other efficacy parameters, the rate of days away from work or school because of infection (2.28 days/patient/year) was relatively low compared to literature reports. [10–14]. Analysis of days on antibiotics revealed that investigators were more likely to treat patients on 21-day infusion schedules with prophylactic antibiotics than patients treated with IVIG every 28 days. This correlation between three week IVIG dosing and prophylactic antibiotic treatment may reflect difficulty in controlling infection in this patient population.

All patients maintained average trough IgG concentrations above the target level of >500 mg/kg with higher mean trough levels observed in recipients of infusions every 21 days compared to patients who received infusions every 28 days. In general, concentration-time profiles were similar for IgG, IgG subclasses and specific antibodies with initial increases on the day of the infusion followed by slow return to baseline at around 14 to 21 days (data not shown).

Half-life results for total IgG and specific antibodies demonstrated significant interpatient variability. The IgG half-life for all patients was approximately 30 days with a 37.5% coefficient of variation. Variation in half-life determinations may reflect shortcomings associated with measuring the decline in concentration of IgG [16]. If the rate of elimination depends on serum concentration, it is possible that half-lives are altered by different doses.

The differences in pharmacokinetic parameters resulted from differences in individual dosing since doses were titrated for each subject. AUC0-t is always larger for the 4-week period compared to the 3-week period unless the dose is reduced, otherwise the patient is receiving more IgG.

Differences in AUC0-t result in differences calculated for CL and Vz. Total body clearance is dose divided by AUC0-t so the difference in AUC0-t produced a difference in total body clearance. Volume of distribution is CL divided by the elimination rate constant. Individual doses were used for calculation of CL. Therefore dose differences also contribute to CL and Vz variation.

Half-life variability may also result from differences in each patient’s ability to synthesize endogenous IgG. If a patient produces IgG, the serum concentration is increased and the half-life appears to be prolonged [16].

Measurement of passively acquired antibodies to specific organisms may provide more meaningful clinical information about IgG metabolism than determination of IgG concentrations by immunochemical tests if the antibodies are not consumed by an infection. Unfortunately antibody levels are extremely low (micrograms or nanograms per mL) compared to IgG (mg/mL) and it is often difficult to accurately measure levels above baseline especially 2–4 weeks after infusion. In this study, determination of the pharmacokinetic parameters for several representative antibodies indicated that antibody activities that are present in the starting plasma are preserved during the manufacture of Biotest-IVIG.
